# Supplementary material for: Clearance of Persistent SARS-CoV-2 RNA Detection in a NFκB-Deficient Patient in Association with the Ingestion of Human Breast Milk: A Case Report
Source: Viruses. 2022 May 13;14(5):1042. doi: 10.3390/v14051042 (PMC9143223; doi:10.3390/v14051042)
Supplement: Supplementary file 1 [file viruses-14-01042-s001.zip › viruses-1700799-supplementary.pdf]

viruses

MDPI

Supplementary Materials

Clearance of Persistent SARS-CoV-2 RNA Detection in a NFκB-Deficient Patient in Association with the Ingestion of Human Breast Milk: A Case Report

Janine S. Sabino [1,†], Mariene R. Amorim [2,†], William M. de Souza [3,†], Lia F. Marega [1], Luciana S. Mofatto [2], Daniel A. Toledo-Teixeira [2], Julia Forato [2], Rodrigo G. Stabeli [4,5], Maria Laura Costa [6], Fernando R. Spilki [7], Ester C. Sabino [8,9], Nuno R. Faria [9,10,11], Bruno D. Benites [12], Marcelo Addas-Carvalho [12], Raquel S. B. Stucchi [13], Dewton M. Vasconcelos [14], Scott C. Weaver [3,15], Fabiana Granja [2,16,†], José Luiz Proenca-Modena [2,17,18,*,†] and Maria Marluce dos S. Vilela [1,*,†]

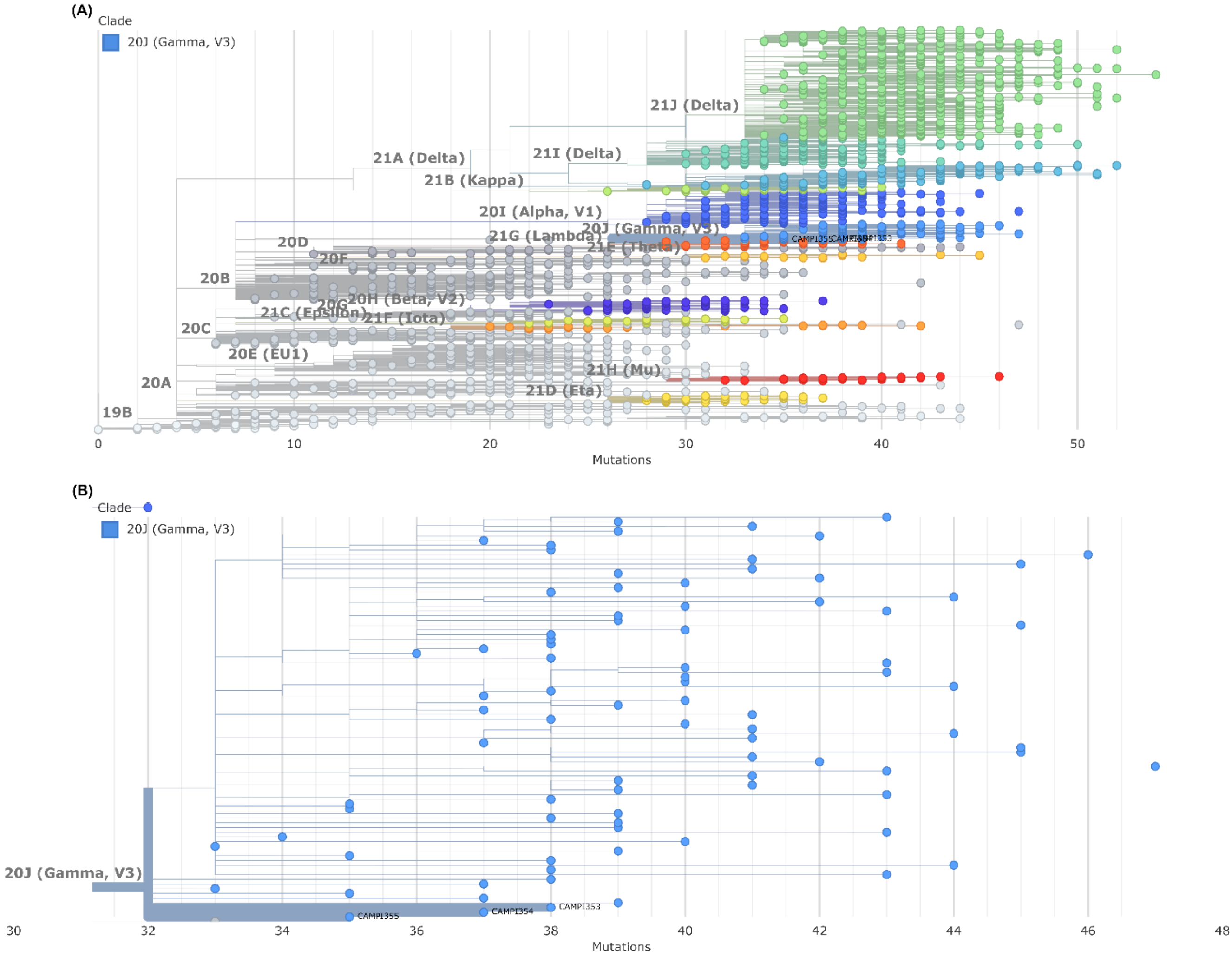

Figure S1. Maximum-likelihood phylogenetic analysis using NextStrain (Auspice tool(17). (**A**) Phylogenetic tree including clades related with 20J (Gamma V3). (**B**) The phylogenetic analysis shows the three SARS-CoV-2 genomes sequences from the proband (i.e., CAMPI353, CAMPI354 and CAMPI355) in the clade 20J (Gamma, V3).

Table S1. Mutation signatures of Gamma (PANGO P.1 lineage or NextStrain clade 20J) from genome sequencing of SARS-CoV-2.

Amino Acid	Protein	Position	CAMPI353 *	CAMPI354 *	CAMPI355 *
aa	*ORF1A*	*S1188L*	confirmed	confirmed	confirmed
aa	*ORF1A*	*K1795Q*	confirmed	confirmed	confirmed
deletion	*ORF1A*	*del3675/3677*	confirmed	confirmed	confirmed
aa	*ORF1B*	*P314L*	confirmed	confirmed	confirmed
aa	*ORF1B*	*E1264D*	confirmed	confirmed	confirmed
aa	*S*	*L18F*	confirmed	confirmed	confirmed
aa	*S*	*T20N*	confirmed	confirmed	confirmed
aa	*S*	*P26S*	confirmed	confirmed	confirmed
aa	*S*	*D138Y*	confirmed	confirmed	confirmed
aa	*S*	*R190S*	confirmed	confirmed	undetermined
aa	*S*	*K417T*	confirmed	confirmed	confirmed
aa	*S*	*E484K*	undetermined	undetermined	undetermined
aa	*S*	*N501Y*	undetermined	undetermined	undetermined
aa	*S*	*D614G*	confirmed	confirmed	confirmed
aa	*S*	*H655Y*	confirmed	confirmed	confirmed
aa	*S*	*T1027I*	confirmed	confirmed	undetermined
aa	*S*	*V1176F*	confirmed	confirmed	confirmed
aa	*ORF3A*	*G174C*	undetermined	undetermined	undetermined
aa	*ORF3A*	*S253P*	undetermined	confirmed	undetermined
aa	*ORF8*	*S84L*	absence	absence	absence
aa	*ORF8*	*E92K*	confirmed	confirmed	confirmed
aa	*N*	*P80R*	confirmed	confirmed	confirmed
aa	*N*	*R203K*	confirmed	confirmed	confirmed
aa	*N*	*G204R*	confirmed	confirmed	confirmed
aa	NSP3	S370L	confirmed	confirmed	confirmed
aa	NSP3	K977Q	confirmed	confirmed	confirmed
aa	NSP3	S1735F	undetermined	undetermined	undetermined
aa	NSP6	L260F	undetermined	undetermined	undetermined
deletion	NSP6	del106/108	confirmed	confirmed	confirmed
aa	NSP12	P323L	confirmed	confirmed	confirmed
aa	NSP13	E341D	confirmed	confirmed	confirmed

* sequences are available on GISAID (accession number: EPI_ISL_6513196, EPI_ISL_6513480, and EPI_ISL_6513725; https://www.gisaid.org). The coding regions are those annotated on Wuhan-Hu-1 (GenBank accession number: NC_045512).

Table S2. Immunophenotyping of our patient.

Cell Types	19 May 2021	12 July 2021	Ref. Values
CD3/mm^3 (%)	849 (88.4)	2673 (91.55)	849.1–1963.3
CD4/mm^3 (%)	234 (27.6)	612 (20.95)	477.5–1140.8
CD8/mm^3 (%)	466 (54.95)	1909 (65.38)	211.7–724.6
CD4/CD8	0.50	0.32	1.2–4.5
CD19/mm^3 (%)	7 (0.7)	0.0	124.2–415.9
CD16$^+$CD56/mm^3	107 (11,1)	158 (5.41)	137–567.8
CD3/CD16$^+$ CD56/mm^3	65 (7.6)	N/P	137–567.8
TCRab$^+$CD3$^+$	760 (89.5)	N/P	-
TCRab$^+$CD3$^+$CD4$^-$CD8$^-$/mm^3	22 (2.9)	N/P	<1.5%
CD4$^+$CD45RA$^+$	N/P	112 (3.83)	137.8–574.5
CD4$^+$CD45RO$^+$	N/P	394 (13.50)	-
CD45$^+$RA$^+$	N/P	1282 (43.90)	-
CD45$^+$RO$^+$	N/P	1413 (48.40)	-

Legend: N/P, not performed.

Table S3. C-reactive protein and ferritin values.

Parameters	19 May 2021	27 May 2021	7 June 2021	22 July 2021	Ref. Values
C-reactive protein (mg/dL)	41.2	1.7	2.0	2.0	<0.5
Ferritin (mg/L)	1464.0	918.7	728.4		30.0–400.0
